# Multispectral Imaging Method for Rapid Identification and Analysis of Paraffin-Embedded Pathological Tissues

**DOI:** 10.1007/s10278-023-00826-9

**Published:** 2023-04-18

**Authors:** Ouafa Sijilmassi, José-Manuel López Alonso, Aurora Del Río Sevilla, María del Carmen Barrio Asensio

**Affiliations:** 1 Faculty of Optics and Optometry, Anatomy and Embryology Department, Universidad Complutense de Madrid, Madrid, Spain; 2 Optics Department, Faculty of Optics and Optometry, Universidad Complutense De Madrid, Madrid, Spain

**Keywords:** Biomedical imaging, Multispectral imaging, Optical absorbance measurement, Endmembers, Principal component analysis

## Abstract

The study of the interaction between light and biological tissue is of great help in the identification of diseases as well as structural alterations in tissues. In the present study, we have developed a tissue diagnostic technique by using multispectral imaging in the visible spectrum combined with principal component analysis (PCA). We used information from the propagation of light through paraffin-embedded tissues to assess differences in the eye tissues of control mouse embryos compared to mouse embryos whose mothers were deprived of folic acid (FA), a crucial vitamin necessary for the growth and development of the fetus. After acquiring the endmembers from the multispectral images, spectral unmixing was used to identify the abundances of those endmembers in each pixel. For each acquired image, the final analysis was performed by performing a pixel-by-pixel and wavelength-by-wavelength absorbance calculation. Non-negative least squares (NNLS) were used in this research. The abundance maps obtained for the first endmember revealed vascular alterations (vitreous and choroid) in the embryos with maternal FA deficiency. However, the abundance maps obtained for the third endmember showed alterations in the texture of some tissues such as the lens and retina. Results indicated that multispectral imaging applied to paraffin-embedded tissues enhanced tissue visualization. Using this method, first, it can be seen tissue damage location and then decide what kind of biological techniques to apply.

## Introduction 

Multi- and hyperspectral imaging are non-destructive techniques that integrate both spectroscopic and imaging techniques or computer vision in one system [1, 2]. These methods are used in numerous medical applications, especially in disease diagnosis and image-guided surgery [3–5]. It is often applied to obtain quantitative information for several biological processes in both healthy and diseased tissues, e.g., in tumor detection and diagnosis, cancer type differentiation and cancer cell metabolism [6–8], and cardiovascular conditions [9, 10], to quantify metabolic activity in cerebral tissue [11, 12] and to measure the changes in the spatial distribution of tissue oxygenation during vascular occlusion [13], among many other applications.

Spectral imaging is based on constructing an image cube which consists of multiple slices of the same scene acquired at a narrow band of the electromagnetic spectrum [2]. The final data acquired by the construction are called “hypercube” since they can be illustrated as containing 3 dimensions with two for spatial coordinates (*x*, *y*) collected in the *X*–*Y* plane and the other one for spectral information *λ* represented in the *Z*-direction [11]. Photon propagation through disordered media such as biological tissues can undergo both scattering (from inhomogeneity of biological structures like extracellular matrix, mitochondria, cell nuclei) and absorption by chromophores such as water, lipids, hemoglobin, or melanin [14]. The relative probability of these processes in a given tissue depends on wavelengths [15].

Folic acid (FA) (vitamin B9) is an essential micronutrient for the growth and development of embryos. Maternal FA deficiency is a serious public health problem [16, 17] and has been implicated in a number of fetal abnormalities, especially, but not exclusively, the nervous system [18]. It is known that during the progression of a disease, the scattering and absorption properties of tissue change [2]. For this reason, in the present work, we aimed to evaluate alterations in the absorbance behavior of embryonic eye tissues due to a maternal folic acid-deficient (FAD) diet by using multispectral analysis.

In previous studies, we have observed that maternal FAD diet alters protein expression patterns in the whole embryonic eye [19, 20], as well as disorganizes fibers/cell orientation [21]. To observe these alterations, before applying image processing, tissues were stained by antibodies to recognize collagen IV laminin-1 in tissue. However, we know that maternal FAD could alter many molecules and tissues of the eye. Traditionally, to analyze pathological tissues, different biological techniques are usually used, among which those that use antibodies for immunocytochemical methods, as well as in situ hybridization experiments that allow for the localization of RNA or DNA targets in cells and tissues. We describe a new imaging technique based on multispectral image analysis applied to paraffin-embedded tissues for non-invasive biomedical diagnosis. The goal of our study was to detect structural alterations of eye tissues as a diagnostic aid before applying other techniques.

## Materials and Method

### Animals and Diet

All mice were housed under temperature- and light-controlled conditions at 22 ± 2 °C on a 12:12 light–dark cycle with free access to food and water. Experiments were carried out in 8-week-old C57/BL/6 J female mice. They were assigned in equal numbers to 3 groups according to the diet: (1) control diet (a standard FA rodent diet, 2 mg/kg diet), (2) FAD diet for 2 weeks (D2 group), and (3) FAD diet for 8 weeks (D8 group). At the conclusion of the experiment, mice were anaesthetized by carbon monoxide and sacrificed by cervical dislocation at 14.5 days of gestation (E14.5). Embryos were removed by cesarean section, placed in cold sterile phosphate-buffered saline, fixed for 48 h in buffered formaldehyde, and decapitated. The heads were then embedded in paraffin following standard procedures. Finally, frontal sections were made with a thickness of 5 µm and placed on slides. Finally, to observe a green autofluorescence without the use of any labels or stains, sections were visualized utilizing a Zeiss Axioplan 2 imaging microscope equipped with a FITC filter. In this study, we studied nine mouse embryos in each group, and one eye per individual was used in the analysis.

The experimental protocol was reviewed and approved by the Animal Experimentation Committee of the Complutense University Madrid (UCM). Mice were maintained in the Animal Facility at the Faculty of Medicine, UCM.

### Description of the Experiment

**Fig. 1:**
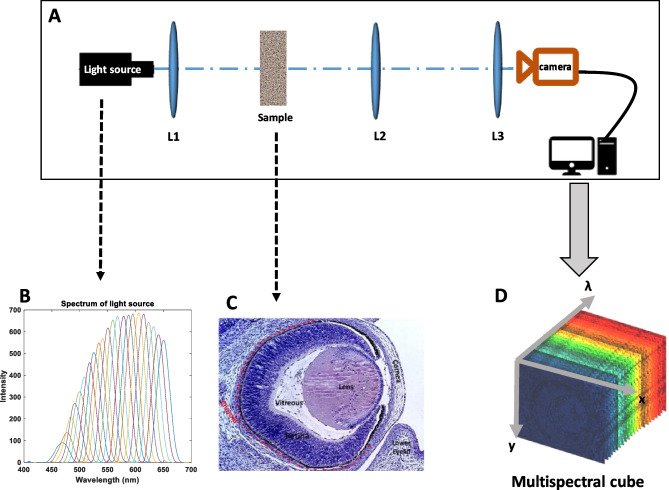
**A** The experimental setup of the proposed method. **B** Spectra of the tunable source from 450 to 657 nm. **C** Frontal section of a control eye stained with hematoxylin–eosin; in this section, it can be seen the different structures of the eye: cornea, lens, vitreous, retina, and choroid. **D** 22-band multispectral data cube

Figure 1A shows the experimental setup used for multispectral image acquisition. In general, the system is made up of the following elements:

Monochromatic illumination for multispectral measurements was provided by a tunable light source TLS-25. Multispectral images were obtained in the spectral range 390–705 nm with a resolution of 25 nm. As shown in Fig. 1B, the spectra have a small bandwidth; besides, the intensity level is different for the different wavelengths. The incident beam passes first through an achromatic doublet lens (L1), to collimate the monochromatic light beam. The emerging light passes through tissue samples; then, light emerging from the tissue is collected and focused on two lenses (L2, *f* = 50 mm; L3, *f* = 400 mm). The acquisition of multispectral images was done using a 1/4″ Sony CCD progressive scan FireWire CCD Color Camera (DFK21AF04); the CCD chip produces a resolution of 640 × 480 pixels per frame. Finally, CCD camera was operated via a FireWire connection to a desktop PC. The software, IC Capture, was used to capture and display image sequences with a frame rate set at 50 frames per second. Once the sample has been focused on, 50 consecutive frames were taken at a certain selected wavelength $\left({\lambda }_{0}\right)$.

Taking into account the spectral sensitivity of the camera, the multispectral images were taken from 390 to 705 nm in 15-nm wavelength steps. The 50 frames were averaged pixel-by-pixel to minimize the temporal effects of noise in CCD detectors. Images were acquired in gray scales from 0 to 256 levels using the Matlab Image Acquisition tool. In general, all the images have been taken in darkness. First, for each wavelength, the image has been taken without the sample and then with it. Therefore, for each wavelength, we have divided the image captured without the sample by the image collected with the sample; in this way, the final image recorded is the transmission of the sample. Finally, we have for each of the 27 eyes analyzed (9 eyes in each sample group) a set of multispectral images $T_{Gi} (x,y,\lambda_{0} )$, where *G* is the group (control, D2, and D8), *i* the analyzed eye (*i* = 1,…, 9), *x*, *y* the pixel in the image, and ${\lambda }_{0}$ is the selected wavelength. The acquired dataset is called spectral data cube (Fig. 1D). The spectral cube combines spatial and spectral information. In total, all captured images form a set of 594 multispectral images. As can be seen, it is a huge amount of information; so, to analyze it, we apply Principal Component Analysis (PCA) method (described below) to reduce the dimensionality of the data.

### Principal Component Analysis Method

Our multispectral images have been analyzed using a multivariate statistical method known as principal component analysis (PCA). It is the best, in the sense of least‐square error, linear dimension reduction technique [22, 23]. It reduces the dimensions of a dataset with minimal loss of information. In essence, PCA seeks to reduce the dimension of the data by performing an orthogonal transformation to the basis of correlation eigenvectors and projects onto the subspace spanned by those eigenvectors corresponding to the largest eigenvalues [24]. In the case of multispectral imaging, PCA re-expresses the original spectra bands by combining their orthogonal linear components with the largest variance. It computes an orthonormal basis for a series of images, transforming the spectra cube to a “noise-less” structure with a lower dimension [14].

We name *F* to a set of frames that can be written as $F = \left\{ {F_{1} ,F_{2} ,...,F_{\lambda } ,...,F_{N} } \right\}$, where *N* is the number of frames chosen registered with a determined spatial frequency. If the detector array camera has *R* rows and *C* columns, it is possible to organize the *M* = *R* × *C* signals of the pixels as a two-dimensional column vector; we can so characterize the position of the pixels, considering its row *r* and its column *c*, as $k = (c - 1)R + r$, where *r* is from 1 to *R* and *c* is from 1 to *C*. Therefore, *k* takes values from 1 to *M*, where *M* is the total number of pixels in the array. Then, a set of *N* frames can be written as an *F* matrix of *M* rows and *N* columns; the matrix F is defined as:

1 $$F = \left( {F_{1} ,F_{2} ,...,F_{N} } \right) = \left[ {\begin{array}{*{20}c} {p_{1} (\lambda_{1} )} & {p_{1} (\lambda_{2} )} & . & {p_{N} (\lambda_{N} )} \\ {p_{2} (\lambda_{1} )} & {p_{2} (\lambda_{2} )} & . & {p_{2} (\lambda_{N} )} \\ . & . & . & . \\ {p_{M} (\lambda_{1} )} & {p_{M} (\lambda_{2} )} & . & {p_{M} (\lambda_{N} )} \\ \end{array} } \right]$$

where $p_{M} (\lambda )$ is the signal of the pixel *M* in the space *λ*. Linear combinations of the original frames form eigenvectors ***E*** of the covariance matrix ***S*** of the original frames. These eigenvectors correspond to the largest eigenvalues $\nu$, known as principal component (PC). The covariance matrix ***S*** is computed by subtracting the sample mean µ of each row of matrix *F* as follows: $S = \frac{1}{n}\sum\limits_{i = 1}^{n} {(F - \mu )(F - \mu )^{T} }$. The expression $(F - \mu )(F - \mu )^{T}$ can be diagonalized by its orthonormal eigenvectors. After diagonalizing ***S*** matrix, we obtain a set of *N* eigenvectors, arranged in an *N* × *N* matrix, ***E***, and *N* eigenvalues, $\nu$, in a process called principal component analysis. This corresponds with the solution of the following eigenvalue equation:

2 $$\left( {S - \nu_{\alpha } I} \right)E_{\alpha } = 0,\begin{array}{*{20}c} {} & {\alpha = 1,2,...,N} \\ \end{array}$$

For all this, the expansion of the PCs is obtained as a *M* × *N* matrix, with the following expression:

3 $$PC = \overline{F} E$$

Each PC, established by columns, is given by:

4 $$PC_{\alpha } = \sum\limits_{\lambda }^{N} {e_{\alpha ,\lambda } \overline{{F_{\lambda } }} }$$

In this way, each PC is an image that is properly combined to produce the original set of frames. Using the eigenvalues to form a new basis, any pixel vector can be expressed as a linear combination of the eigenvectors:

5 $$\overline{F} = PC \times E^{T}$$

This leads us to the following equation:

6 $$\overline{{F_{\lambda } }} = \sum\limits_{\alpha }^{N} {e_{\lambda ,\alpha } \times PC_{\alpha } }$$

The element $e_{\lambda ,\alpha }$ is the weight of the PC in the frame $F_{\lambda }$.

### Calculation of the Absorbance or Optical Density

For a given wavelength $\left({\lambda }_{0}\right)$, the absorbance of a given sample can be defined as:

7 $$A_{Gi} \left( {x,y,\lambda_{0} } \right) = - \ln \left( {T_{Gi} \left( {x,y,\lambda_{0} } \right)} \right)$$

where $T_{Gi} \left( {x,y,\lambda_{0} } \right)$ is the transmission of the sample at a defined wavelength; it can be defined as follows:

8 $$T_{Gi} \left( {x,y,\lambda_{0} } \right) = e^{{ - \sum\limits_{j = 1}^{n} {\varepsilon_{j} (\lambda_{0} ).C_{j} (x,y).L} }}$$

In this way, the absorbance defined in (7) will be written as:

9 $$A_{Gi} \left( {x,y,\lambda_{0} } \right) = \sum\limits_{j = 1}^{n} {\varepsilon_{j} (\lambda_{0} ) \times C_{j} (x,y) \times L}$$

where ${\varepsilon }_{j}$ is the total absorption coefficient of a given component *j* present in the sample, ${\varepsilon }_{j}(x,y)$ is the concentration of *j* at the image point (*x*, *y*), and *L* is the thickness of the sample that, in our case, is 5 µm.

If now we apply a decomposition of principal components onto absorbance data, where the average value of each absorbance image $\left\langle {A_{Gi} (\lambda_{0} )} \right\rangle_{x,y}$ was removed to obtain images with zero mean, so we would have:

10 $$A_{Gi} (x,y,\lambda_{0} ) - \left\langle {A_{Gi} (\lambda_{0} )} \right\rangle_{x,y} = \sum\limits_{\alpha = 1}^{N} {e_{\alpha } (\lambda_{0} ) \times PC_{\alpha } (x,y)}$$

**Fig. 2:**
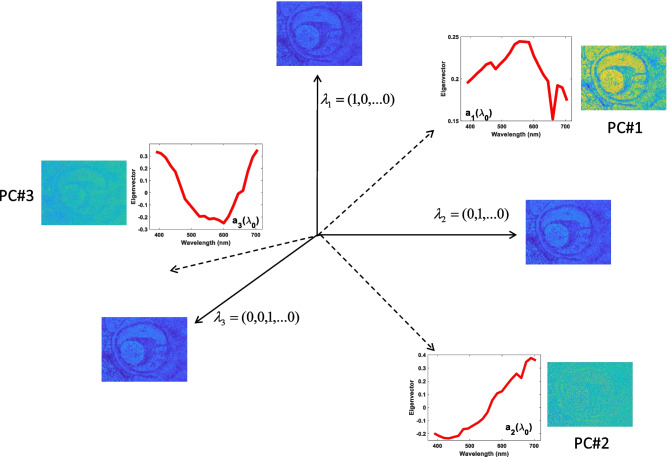
An example of decomposition in PCs. The eigenvectors (in dashed line) designate ways to mix the original images (at the end of the solid lines) to obtain uncorrelated images (PCs). PCs are linear combinations of the original images. The coefficients of these linear combinations are the components of the eigenvectors

This decomposition interprets each of the images as a mixture of original images uncorrelated between them. These images are the PCs. Since the different images were taken at different wavelengths, the eigenvectors $e_{\alpha }$ are those that carry the trend in $\lambda_{0}$ and explain how we must blend the principal components to obtain the initial images, while the PCs carry the spatial dependence. Then, if we compare (9) with (10), we see that eigenvectors are related to the absorption coefficients $\varepsilon_{j}$ and the PCs to the spatial distribution of said component. The eigenvalues represent the total variance of each PC. Figure 2 shows the original images located in a configuration space, where each direction symbolizes a certain *λ*. In bold, there are three axes associated with *λ*1, *λ*2, and *λ*3 taken, which are therefore associated with the directions (1, 0…0), (0, 1…0), and (0, 0, 1… 0), respectively. Next to these axes appear the absorbance images taken for each of these wavelengths. The images have a non-zero covariance since they show great similarity between them. The decomposition of the CPs can be seen as a search for new directions in this space (eigenvectors), such that when the original images are mixed, taking the components of the eigenvectors as coefficients of the linear combination, new images are obtained (PCs) that show a null correlation between them. These new principal directions (eigenvectors) appear as dotted lines in the figure. The eigenvector and PC associated with that direction are represented next to them. Lastly, the eigenvalue would represent the variance of the PC associated with that direction.

The analysis of the PCs involves decomposing the absorbance images according to the following decomposition:

11 $$A(x,\lambda_{0} ) = \left\langle {A(\lambda_{0} )} \right\rangle + \sum\limits_{\alpha } {e_{\alpha } (\lambda_{0} ) \times PC_{\alpha } (x)}$$

where *x* is a spatial coordinate within the image, ${\lambda }_{0}$ is the wavelength, ${e}_{\alpha }\left({\lambda }_{0}\right)$ is the eigenvector, and ${PC}_{\alpha }\left(x\right)$ is the image corresponding to the PC_α_.

This decomposition makes all Pcs have a zero mean as well as can contain both positive and negative values. Likewise, eigenvectors are difficult to interpret directly as spectra, since they also have positive and negative values that reflect values above or below the mean absorbance, $A(\lambda )$. For this reason, the need has arisen in the hyperspectral image analysis environment to carry out a decomposition analogous to that described by Eq. (11), but in the following way:

12 $$A(x,\lambda ) = \sum\limits_{k} {E_{k} (\lambda ) \times Ab_{k} (x)}$$

where ${E}_{k}\left(\lambda \right)$ is called “endmember *k*” generally defined as a spectrally unique, idealized, and pure signature spectrum weighted by their correspondent fractions, or abundances ${Ab}_{k}(x)$. The pure spectral signature signifies the complete reflectance of a pixel exclusively occupied by single surface material. These pure signatures can decompose the hyperspectral scene into abundance fractions by means of spectral unmixing algorithms [25]. Many endmember extraction algorithms have been developed including pixel purity index [26], N-FINDR algorithm [27], and the orthogonal subspace projection (OSP) algorithm [28].

In the current study, Eq. (12) involves solving not a single system of equations, but one for each point *x* in the image, with the condition that the abundances must be numbers greater than zero. This non-negative least square (NNLS) algorithm is subject to all the solutions being greater than or equal to zero. Mathematically, obtaining the abundances is equivalent to solving the system $Ux = b$, for each point *x*, where *b* is the regression vector of the least squares problem; hence, *x* would be calculated as $x = U^{ - 1} b$. However, the *U*-matrix, in our case the collection of endmembers *k*, is generally not a square matrix (the number of wavelengths is usually greater than the number of spectra considered relevant); hence, it only makes sense to talk about solving the system approximately by using pseudo-inverses. That is, we use the formula $U^{T} \times U \times x = U^{T} \times best$ with the following solution $x = \left( {U^{T} U} \right)^{ - 1} U^{T} best$, which is not exact since *best* is an approximation of *b* [29]. Furthermore, the solution is restricted to considering only values in which “*x*” takes positive values. This algorithm is known as NNLS and is fast and requires a small amount of computer memory. In this work, NNLS implemented in the open-source Matlab Hyperspectral Toolbox has been used [30].

**Fig. 3:**
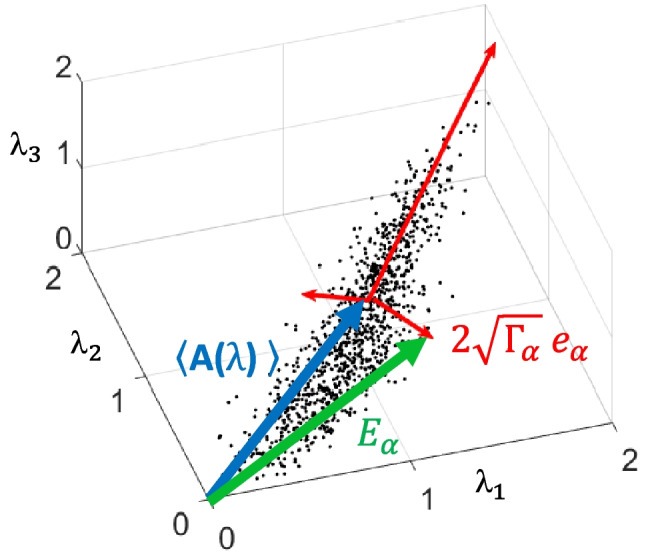
Point cloud diagram representation (scatter plot) of the pixels of three absorbance images, together with the vector representing the mean absorbance $\left\langle {A(\lambda )} \right\rangle$ (blue arrow), close to the eigenvectors of the principal directions ${e}_{\alpha }$ (red arrow). Finally, in green color, we draw the representation of the formation of the endmembers

The initial problem of calculating the abundance is to find the spectra to be considered endmember. In this study, we used the structure of the eigenvectors associated with the PCs to obtain endmembers. As can be seen in Fig. 3, the pixel values of the absorbance of three images of a control eye at three wavelengths $\left({\lambda }_{1}=435nm,{\lambda }_{2}=540nm,{\lambda }_{3}=660nm\right)$ have been represented in a “scatter plot” diagram. The directions associated with the eigenvectors of that “scatter plot” have also been represented; their length is proportional to data propagation in these directions. The standard deviation of this dispersion is given by $\sqrt {\Gamma_{\alpha } }$, where ${\Gamma }_{\alpha }$ is the eigenvalue associated with the direction *α*; note that eigenvectors have a unit module. Hence, if we form the vector $E_{\alpha } (\lambda ) = A(\lambda ) + 2\sqrt {\Gamma_{\alpha } } e_{\alpha } (\lambda )$, we are forming a spectrum that would be represented as a pixel located at the ends of the red arrows shown in Fig. 3. These new vectors continue to be related to the eigenvectors and, at the same time, represent spectra located on the “outside” of the point cloud that forms the scatter plot. The new vectors also satisfy the conditions to be considered endmembers.

After obtaining the endmembers ${E}_{\alpha }$, the NNLS algorithm was used to estimate the corresponding abundances $Ab(x)$ for each of them as a positive number that we can directly relate to the concentration of the said spectrum in the image. Given the orthogonality of the eigenvectors from which the endmembers come, they will reflect structures in the image as disjoint as possible and ordered in order of importance in the data [31].

## Results

**Fig. 4:**
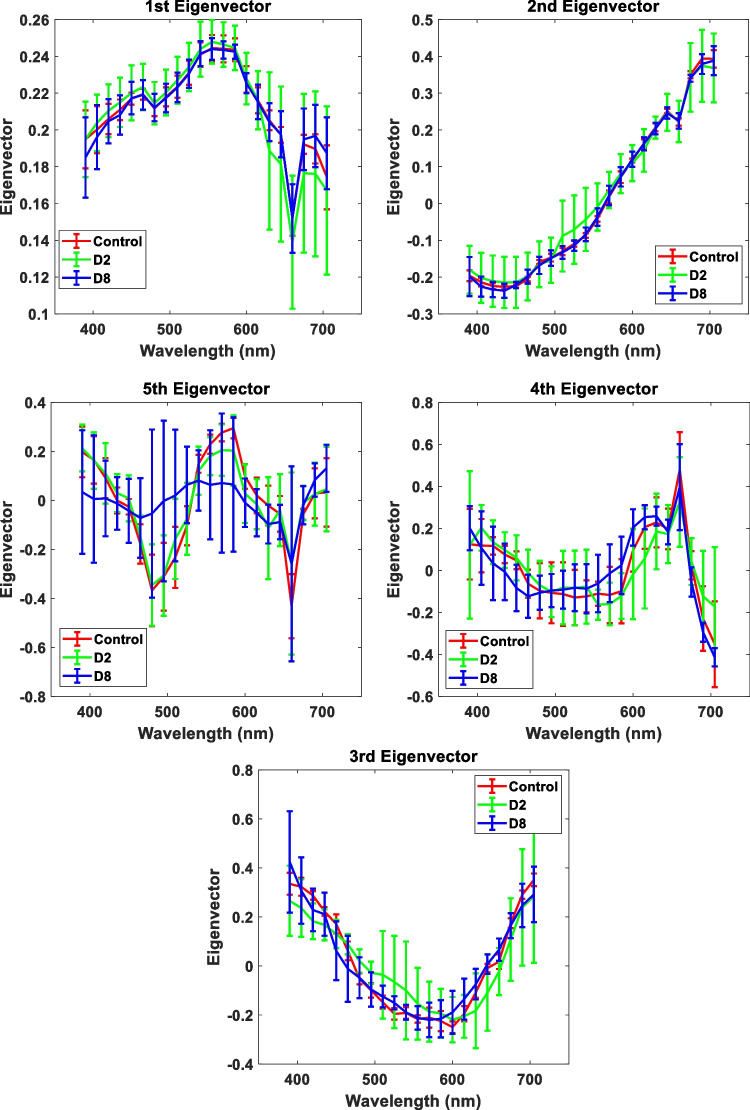
Eigenvectors associated with PCs # 1, 2, 3, 4, and 5, for the three groups, control, D2, and D8, averaged over all eyes of each group

Table 1 shows the eigenvalues and percentages of the variance associated with each component and for each sample group (control, D2, and D8). As can be seen, in all groups studied, there are five eigenvalues, in order of importance, that accounted for greater than 99% of the total variance. On the other hand, Fig. 4 shows the mean value of the eigenvectors, from #1 to #5, together with its errors. From this figure, it can be deduced that each eigenvector is approximately the same for all eyes that comprise the same group since the error bars are small. For this reason, their mean values will be used as a representative eigenvector base of the entire sample group.

**Table 1:** Eigenvalues and percentages of variance associated with each principal component

# PC	Eigenvalue			Percentage of variance (%)		
	Control	D2	D8	Control	D2	D8
1	2.41	1.83	1.93	88.08	85.63	86.79
*2*	0.25	0.24	0.19	9.27	11.04	8.65
*3*	0.03	0.03	0.06	1.25	1.39	2.51
*4*	0.02	0.01	0.02	0.56	0.65	0.79
*5*	0.01	0.01	0.01	0.33	0.42	0.49

The error bars most often represent the standard deviation of a dataset. We have observed that within the same individuals of the two groups D2 and D8, the standard deviations were large which indicates that there are a lot of variances in the observed data, that is, there is wide variation among individuals of the same group. As can be seen, the eigenvectors are quite similar but with a slight difference in the spectral behavior between the control and the FAD groups.

**Fig. 5:**
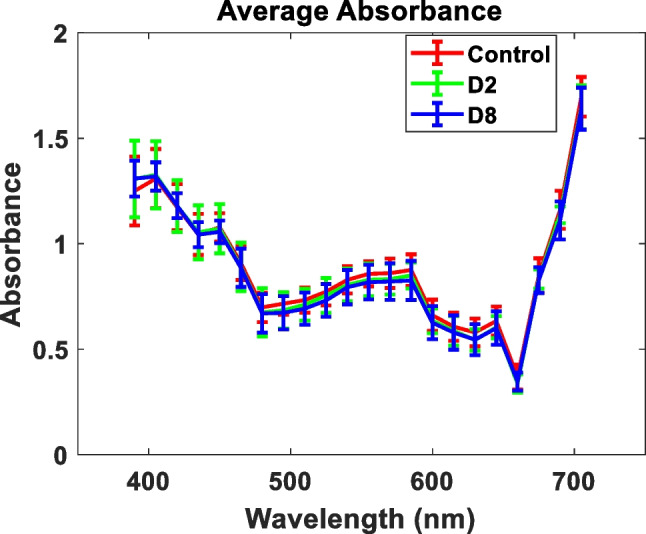
The average absorbance for all groups of images studied

Finally, we represent in Fig. 5 the mean absorbance $\left\langle {A_{Gi} (\lambda_{0} )} \right\rangle_{x,y}$ for all groups. As can be seen, the wavelength most absorbed was around 420 nm.

**Fig. 6:**
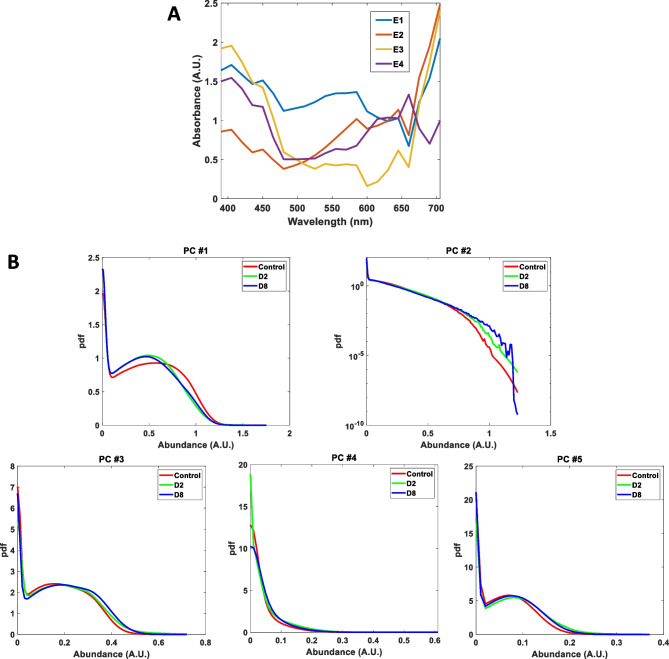
**A** Endmembers of the control group. **B** Mean probability distribution of the abundance maps for each endmember studied, for the eyes of the control, D2, and D8 groups

In our case, we have found five relevant endmembers (Fig. 6A). After the endmembers have been found, NNLS algorithm was used to obtain abundance maps of all groups. The abundance map identifies the proportion of each endmember present in the spectra of each pixel. To analyze these maps, first, we study the probability density function (pdf) of the abundance maps of each endmember; the results appear in Fig. 6B. In this figure, it can be seen how the highest abundances are for endmember #1 (first component) and endmember #3 (third component).

**Fig. 7:**
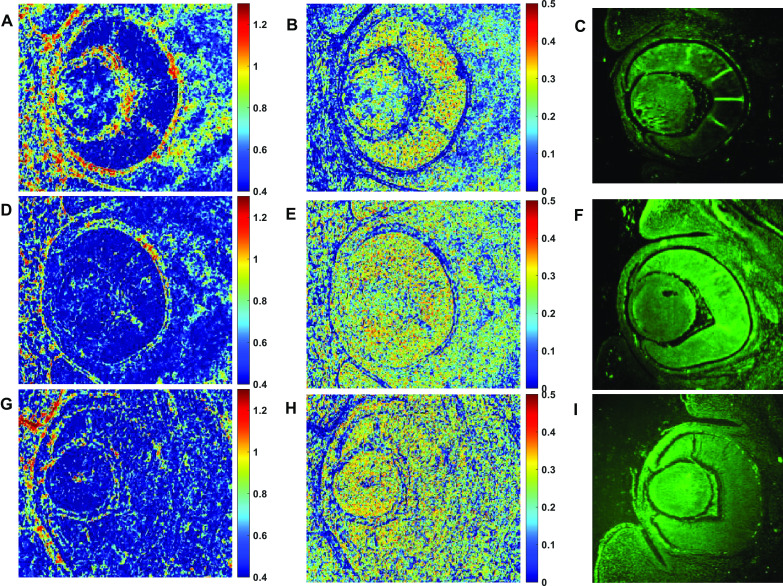
Abundance maps for endmembers # 1 (**A**, **D**, and **G**) and # 3 (**B**, **E**, and **H**) of one eye from each group. The autofluorescence images of the same paraffin-embedded tissue samples **(C**, **F**, and **I**), without the use of any labels or stains. Magnification × 10 objective

Images of the abundance maps are detailed in Fig. 7. Abundance maps are images of the abundance values shown across all pixels in the image. These maps show detailed data for one individual. Figure 7A, D, and G represent the abundance maps of the first endmember for control, D2, and D8 individuals, respectively.

**Fig. 8:**
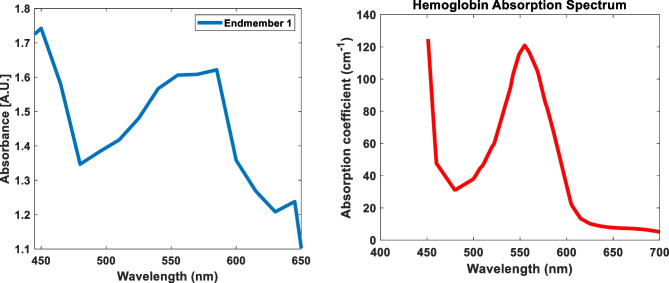
Comparison between the first endmember and the absorption coefficient of hemoglobin

In Fig. 7A (control), it can be seen how the spectrum is mainly concentrated in the vitreous and the choroid, indicating especially the areas of blood vessels (to distinguish the different structures of the eye, see Fig. 1C). However, these points of high abundance decrease in the abundance maps of both groups D2 and D8, especially in the vitreous region. The areas where there is the greatest difference are 450–500 nm, a peak at 550 nm, and a drop from 600 nm. Of all chromophores that are present in the eye and can exhibit this type of behavior is maybe the hemoglobin (Fig. 8). As can be seen, both spectra are similar.

On the other hand, the abundance maps obtained from the third endmember (Fig. 7 (central column)) seem associated with both structures the retina and lens, since there is the presence of high abundances greater than 0.2.

## Discussion

This paper presents the findings of a multispectral analysis, based on the measured spectral difference between normal embryonic eye tissues and tissues obtained from embryos with a maternal FA deficiency diet. Results of the first abundance maps suggest that eyes from D2 and D8 groups exhibited alterations in the vascular region of both vitreous and choroid. It is known that FA deficiency affects directly red blood cell metabolism and oxygen balance that they can carry to the tissues [32]. In fact, low oxygen content in blood, due to a deficit of FA, triggers a whole series of adaptive responses by the body, especially, reduction of cell proliferation and change in the size of the lumen of the blood vessels, as well as changes in the shape of red blood cells (they tend to deform tending to a spherical shape) [33, 34]. Therefore, those alterations should be observed in those areas where it is normal to see blood vessels, such as vitreous and choroid. These areas are precisely those that appear signaled in the images of differences in spectral behavior between the control and the groups D2 and D8. In summary, we could link the changes detected to an imbalance in the levels of hemoglobin/oxyhemoglobin due to maternal FA deficiency. Vascular pattern alteration observed in the vitreous body and choroid could be triggered by the hypoxia induced by FA deficiency. In recent research [35], we have observed that the size and density of choroidal vessels (which lies under the retinal pigment epithelium) and hyaloid blood vessels (located within the vitreous) were increased in various embryos from both groups D2 and D8. Specially, we found large choroidal vessel caliber than those of the control group; this abnormality was the cause of retinal detachments in those embryos. In conclusion, our method was able to detect changes in blood vessels only by evaluating paraffin-embedded tissues (unstained) by using multispectral analysis.

On the other hand, the abundance maps obtained from the third endmember indicate some changes in the texture within the cornea, lens, retina, and eyelids. Maybe they are associated with microstructural alterations like a change in the spatial organization of the extracellular matrix as well as small changes in the fiber orientation. All these texture alterations mentioned were found in previous studies realized by our research group [20, 21, 36]. Furthermore, a strong green autofluorescence can be observed in tissues from D2 and D8 groups compared to the control. The autofluorescence in the tissue was studied without the use of any exogenous fluorophores. In another recent study [37], we also observed alterations at the lens, retina, vitreous, and choroid levels, using polarized light applied to these tissues.

The present study clearly shows that the optical characterization of tissues by multispectral imaging can be used efficiently to discriminate pathological tissues embedded in paraffin since the findings in both studies would seem to concur. It is known that absorption peaks at a specific wavelength can be related to a particular molecule. These peaks could be used as a fingerprint of the molecules’ response to light, providing valuable information that can be becoming useful for diagnosis purposes [38]. Our method reveals important information about structural and/or biochemical changes in the different components of ocular structures, which indicates that this novel technique can serve as a useful tool for studying cellular or molecular alterations in tissues before the applications of molecular labeling.

## Conclusions and Future Research

Multi- and hyperspectral imagery are well-known biomedical imaging techniques; both have in common that light-tissue interaction is used as a source of information since the acquired data contain spectral information about optical properties of the tissue such as absorption and scattering. We describe a novel method that combines multispectral imaging in the visible spectrum, data postprocessing, and reconstruction methods, which allows estimating tissue optical density for each point in the image at multiple wavelengths of light. Statistical analysis was computed using the PCA method, where the original multispectral dataset was transformed via PCA from its wavelength space into a new dimensional space. The main advantage of this technique is that the sample preparation is eliminated, that is, non-deparaffinized tissues should be used.

The method has been tested on normal and pathological eye tissues and showed reasonably good results. However, the authors are aware of the fact that more types of tissues are needed for testing the method adequately. Furthermore, new more extended studies should be carried out in the future to evaluate the kind of devices and parameters that can be used to further elucidate these findings before this technique is recognized as an applicable method of non-deparaffinized tissue assessment.

## References

[1] Barbin DF, Sun D-W, Su C (2013). NIR hyperspectral imaging as non-destructive evaluation tool for the recognition of fresh and frozen–thawed porcine longissimus dorsi muscles. Innov Food Sci Emerg Technol.

[2] Balas C, Pappas C, Epitropou G: Multi/hyper-spectral imaging. Handbook of Biomedical Optics:131–164, 2011

[3] Lu G, Fei B (2014). Medical hyperspectral imaging: a review. J Biomed Opt.

[4] Tate TH (2016). Multispectral fluorescence imaging of human ovarian and fallopian tube tissue for early-stage cancer detection. J Biomed Opt.

[5] Ortega S (2020). Hyperspectral imaging for the detection of glioblastoma tumor cells in H&E slides using convolutional neural networks. Sensors.

[6] Akbari H, Uto K, Kosugi Y, Kojima K, Tanaka N (2011). Cancer detection using infrared hyperspectral imaging. Cancer Sci.

[7] Aboughaleb IH, Aref MH, El-Sharkawy YH (2020). Hyperspectral imaging for diagnosis and detection of ex-vivo breast cancer. Photodiagnosis Photodyn Ther.

[8] Ma L, Little JV, Chen AY, Myers L, Sumer BD, Fei B (2022). Automatic detection of head and neck squamous cell carcinoma on histologic slides using hyperspectral microscopic imaging. J Biomed Opt.

[9] Chin JA, Wang EC, Kibbe MR (2011). Evaluation of hyperspectral technology for assessing the presence and severity of peripheral artery disease. J Vasc Surg.

[10] Bruins AA, Geboers DG, Bauer JR, Klaessens JH, Verdaasdonk RM, Boer C (2021). The vascular occlusion test using multispectral imaging: a validation study: the VASOIMAGE study. J Clin Monit Comput.

[11] Giannoni L, Lange F, Tachtsidis I (2018). Hyperspectral imaging solutions for brain tissue metabolic and hemodynamic monitoring: past, current and future developments. Journal of Optics.

[12] Giannoni L: Hyperspectral imaging of the haemodynamic and metabolic states of the exposed cortex. UCL (University College London) 2020

[13] Zuzak KJ, Schaeberle MD, Lewis EN, Levin IW: Visible spectroscopic imaging studies of normal and ischemic dermal tissue. Proc. Biomedical Spectroscopy: Vibrational Spectroscopy and Other Novel Techniques: City

[14] Boas DA, Pitris C, Ramanujam N: Handbook of biomedical optics: CRC Press, Boca Raton, 2016

[15] Patterson MS, Wilson BC, Wyman DR: The propagation of optical radiation in tissue. II: Optical properties of tissues and resulting fluence distributions. Lasers Med Sci 6:379–390, 1991

[16] Wald N, Sneddon J, Densem J, Frost C, Stone R (1991). Prevention of neural tube defects: results of the Medical Research Council Vitamin Study. Lancet.

[17] Botto LD (2006). Trends of selected malformations in relation to folic acid recommendations and fortification: an international assessment. Birth Defects Research Part A: Clinical and Molecular Teratology.

[18] Balashova OA, Visina O, Borodinsky LN (2018). Folate action in nervous system development and disease. Dev Neurobiol.

[19] Sijilmassi O, López Alonso JM, Barrio Asensio MC, Del Río Sevilla A (2018). Collagen IV and laminin-1 expression in embryonic mouse lens using principal components analysis technique. J Microsc.

[20] Sijilmassi O, López Alonso J-M, Del Río Sevilla A, Barrio Asensio MdC: Multifractal analysis of embryonic eye structures from female mice with dietary folic acid deficiency. Part I: Fractal dimension, lacunarity, divergence, and multifractal spectrum. Chaos, Solitons & Fractals 138:109885, 2020

[21] Sijilmassi O, López-Alonso JM, Barrio Asensio MDC, Del Río Sevilla A: Alteration of lens and retina textures from mice embryos with folic acid deficiency: image processing analysis. Graefe’s Archive for Clinical and Experimental Ophthalmology:1–13, 2018 10.1007/s00417-018-4176-5 30392021

[22] Jackson JE: A user’s guide to principal components: John Wiley & Sons, 2005

[23] Jolliffe IT: Principal component analysis: Springer-Verlag, New York, 2002

[24] Kambhatla N, Leen TK (1997). Dimension reduction by local principal component analysis. Neural Comput.

[25] Kale KV, Solankar MM, Nalawade DB: Hyperspectral Endmember Extraction Techniques: IntechOpen, 2019

[26] Boardman JW, Kruse FA, Green RO: Mapping target signatures via partial unmixing of AVIRIS data, 1995

[27] Winter ME: N-FINDR: An algorithm for fast autonomous spectral end-member determination in hyperspectral data. Proc. Imaging Spectrometry V: City

[28] Harsanyi JC, Chang C-I (1994). Hyperspectral image classification and dimensionality reduction: An orthogonal subspace projection approach. IEEE Transactions on geoscience and remote sensing.

[29] Bro R, De Jong S (1997). A fast non-negativity-constrained least squares algorithm. Journal of Chemometrics: A Journal of the Chemometrics Society.

[30] Gerg I, Kun D: Matlab hyperspectral toolbox, 2012

[31] Van der Meer FD, Jia X (2012). Collinearity and orthogonality of endmembers in linear spectral unmixing. International Journal of Applied Earth Observation and Geoinformation.

[32] De Bruyn E, Gulbis B, Cotton F (2014). Serum and red blood cell folate testing for folate deficiency: new features?. Eur J Haematol.

[33] Mozos I: Mechanisms linking red blood cell disorders and cardiovascular diseases. BioMed research international 2015, 2015 10.1155/2015/682054 PMC4331396 25710019

[34] Verduzco LA, Nathan DG (2009). Sickle cell disease and stroke. Blood.

[35] Sijilmassi O, Del Río Sevilla A, Maldonado Bautista E, Barrio Asensio MdC: Gestational folic acid-deficiency alters embryonic eye development: possible role of basement membrane proteins in eye malformations. Nutrition:111250, 2021 10.1016/j.nut.2021.111250 33962364

[36] Sijilmassi O, López Alonso J-M, Del Río Sevilla A, Barrio Asensio MdC: Multifractal analysis of embryonic eye tissues from female mice with folic acid deficiency. Part II: Local Connected Fractal Dimension Analysis. Chaos, Solitons & Fractals 138:109887, 2020

[37] Sijilmassi O, López‐Alonso JM, Del Río Sevilla A, Barrio Asensio MdC: Development of a polarization imaging method to detect paraffin‐embedded pathology tissues before applying other techniques. Journal of biophotonics:e202000288, 2020 10.1002/jbio.202000288 32981228

[38] Ortega S, Halicek M, Fabelo H, Callico GM, Fei B (2020). Hyperspectral and multispectral imaging in digital and computational pathology: a systematic review. Biomedical Optics Express.

